# Population‐level lateralization of boxing displays enhances fighting success in male Great Himalayan leaf‐nosed bats

**DOI:** 10.1002/ece3.9879

**Published:** 2023-03-08

**Authors:** Chunmian Zhang, Jeffrey R. Lucas, Jiang Feng, Tinglei Jiang, Congnan Sun

**Affiliations:** ^1^ Key Laboratory of Animal Physiology, Biochemistry and Molecular Biology of Hebei Province, College of Life Sciences Hebei Normal University Shijiazhuang China; ^2^ Department of Biological Sciences Purdue University West Lafayette Indiana USA; ^3^ Jilin Provincial Key Laboratory of Animal Resource Conservation and Utilization Northeast Normal University Changchun China; ^4^ College of Life Science Jilin Agricultural University Changchun China

**Keywords:** aggressive display, bats, behavioral lateralization, boxing

## Abstract

Behavioral lateralization with left‐ and right‐hand use is common in the Animal Kingdom and can be advantageous for social species. The existence of a preferential use of the hands during agonistic interactions has been described for a number of invertebrate and vertebrate species. Bats compose the second largest order of mammals. They not only use their forelimbs for flight but also agonistic interactions. However, whether bat species show a population‐level lateralized aggressive display has largely been unexplored. Here, we examine the lateralization of boxing displays during agonistic interactions in male Great Himalayan leaf‐nosed bats, *Hipposideros armiger*, from three different populations. We found a population‐level lateralization of boxing displays: Males from all three populations show a preferential use of the left forearm to attack opponents. In addition, left‐handed boxers have higher fighting success over right‐handed boxers. This study expands our knowledge of the handedness of bats and highlights the role of behavioral lateralization in conflict resolution in nocturnal mammals.

## Graphical abstract

Male Great Himalayan leaf‐nosed bats show a population‐level lateralized aggressive display, which performs with a left forearm bias when using forearms for fighting, and left‐handed boxers won more contests than right‐handed boxers. This is the first experimental evidence of lateralization of aggressive displays in a bat species.

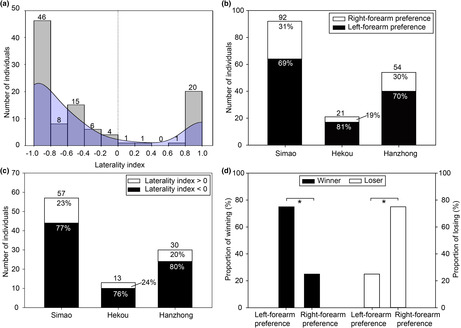

## 1 INTRODUCTION

Behavioral lateralization refers to the preferential side use of limbs and sensory organs and is common in the Animal Kingdom (Rogers, Rigosi, et al., 2013; Rogers, Vallortigara, & Andrew, 2013). Behavioral asymmetries occur in many animals as a consequence of brain specialization and can be advantageous by increasing processing efficiency in reaction times to novel stimuli and improving efficiency when multi‐tasking (Rogers et al., 2004; Rogers, Rigosi, et al., 2013; Rogers, Vallortigara, & Andrew, 2013; Vallortigara & Rogers, 2005).

Populations are said to be lateralized (i.e., directional lateralization) when more than half of the individuals in a population are lateralized in the same direction (Rogers, 2002). Population‐level lateralization is commonly exhibited in social species during their foraging behavior, fear responses, and aggressive behaviors (Rogers, 2002; Vallortigara & Rogers, 2005). Population‐level biases can be evolutionarily stable as illustrated in the competition–coordination model (Ghirlanda et al., 2009). This model predicts that minority‐type individuals occur because they display unexpected fighting behaviors that rivals are less accustomed to during competitive interactions. Nonetheless, majority‐type individuals gain a fitness advantage during interactions that require coordination, such as when mating. For example, minority‐type giant Australian cuttlefish (*Sepia apama*) with a right‐eye preference had higher success during fighting interactions, and majority‐type individuals with a left‐eye preference had higher success during mating interactions (Schnell et al., 2019).

Aggressive displays during agonistic interactions play a crucial role in conflict resolution because they allow individuals to assess their own or their opponent's resource‐holding potential (RHP; defined as “fighting ability”) thus facilitating withdrawal decisions of contests without resulting in physical harm (Bradbury & Vehrencamp, 2011). Population‐level behavioral lateralization in limb use during antagonistic interactions has been described in invertebrates such as blowflies (Romano et al., 2015), olive fruit flies (Benelli, Romano, et al., 2015), and honeybees (Rogers, Rigosi, et al., 2013; Rogers, Vallortigara, & Andrew, 2013), and in vertebrates (reviewed in Rogers, 2009) such as toads (Robins et al., 1998), lizards (Deckel, 1995), chicks (Howard et al., 1980), pigs (Camerlink et al., 2018), fallow deer (Jennings, 2014), gelada baboons (Casperd & Dunbar, 1996) and humans (Richardson & Gilman, 2019).

To expand our understanding of behavioral bias in animal behavior, it is essential to explore the existence and the advantage of laterality during agonistic interactions in a broad range of vertebrates. Bats (Chiroptera) are unique mammals capable of flight and constitute the second largest radiation in mammals, with over 1456 species worldwide (Simmons & Cirranello, 2022). Echolocating bats not only emit echolocation pulses for navigation and prey acquisition but also communicate with a rich assortment of social vocalizations accompanied by behavioral displays (Chaverri et al., 2018). For example, many bats are involved in daily agonistic displays that function in territory defense or competition for mates. These displays included broadband calls, pushing, wing flapping, and boxing moves (Clement & Kanwal, 2012; Fernandez et al., 2014; Prat et al., 2016). While Schreiber's Long‐Fingered bat (*Miniopterus schreibersii*) displays left forelimb lateralization during climbing (Zucca et al., 2010), the potential for population‐level lateralized aggressive behavior remains uncertain.

Here, we examine population‐level lateralization in aggressive displays in the male Great Himalayan leaf‐nosed bat, *Hipposideros armiger*, and test whether minority‐type or majority‐type lateralization enhances fighting success. *H. armiger* is a nocturnal and highly social species that usually roosts in caves, sharing day and night roosts among hundreds of individuals. Our previous studies showed that adult males defend their small, private roosting territory using conspicuous aggressive displays, e.g., boxing (Sun et al., 2019, 2022). Boxing is exhibited during escalated physical combat between two opponents, and males typically use only one forearm to knock each other until one of them retreats (Zhang, Sun, Lucas, Feng, & Jiang, 2022; Zhang, Sun, Lucas, Gu, et al., 2022; Video S1). We hypothesized that a population‐level lateralized boxing behavior would be displayed by male *H. armiger*. We predicted that there would be a significant difference in the proportion of a preferential use of the left or right forearms during agonistic interactions with a bias toward left‐forearm use. If a population‐level lateralized boxing behavior is detected, we further hypothesized that minority‐type males would have an advantage in territorial conflict resolution based on the competition–coordination model. Therefore, we predicted that minority‐type males will be more successful in fights than majority‐type males.

## 2 METHODS

### 2.1 Data collection

Two data sets were used in this study. For the first data set, we reanalyzed video recordings of dyadic agonistic interactions between male *H. armiger* originally described in Sun et al. (2019). Briefly, Sun et al. (2019) captured 230 adult males from Hanzhong (96), Simao (88), and Hekou (46) in August–November 2017. Eight bats were selected randomly and were introduced into a testing cage (0.5 m × 0.5 m × 0.5 m) to trigger agonistic interactions within each trial (Video S2). Trials were monitored until the first interaction between two of the eight bats terminated with a clear winner and loser. Otherwise, trials were terminated if no aggressive interaction occurred within 15 min. Only one aggressive interaction was allowed to occur in each trial. After the trial, we removed all bats and reintroduced another eight bats into the testing cage. Taken together, 115 agonistic interactions from 230 individuals were analyzed.

The second data set comes from Zhang, Sun, Lucas, Gu, et al. (2022) in which 96 adult males from Simao were caught in July–August 2019. There was no overlap between the Simao bats tested in Sun et al. (2019) compared with the bats tested in Zhang, Sun, Lucas, Gu, et al. (2022) because they were captured at different roosting cave sites. Indeed, the distance between these two caves was at least 20 km and it is very likely that they were isolated from one another. Agonistic interactions were performed between pairs in a box made of acrylic sheet plexiglass (long × wide × high: 1 m × 0.5 m × 0.5 m). Pairs of bats were chosen at random and placed in the center of the two pieces of wire mesh on opposite ends of a slide rail. The experimenter pulled the two pieces of wire mesh toward each other by means of ropes until the two bats arrived at the center of the box; this is a simulation of two bats invading each other's territory (Video S1). The criteria for termination of the experiment were the same as the criteria from Sun et al. (2019). Taken together, 48 agonistic interactions from 96 individuals were analyzed from the Zhang, Sun, Lucas, Gu, et al. (2022) study.

For the above two studies, each male was tested in only one agonistic interaction to avoid pseudoreplication. All of the agonistic interactions were monitored using night‐shot camcorders (FDR‐AX60, Sony Corp.). The experimental procedures are described in detail by Sun et al. (2019) and Zhang, Sun, Lucas, Gu, et al. (2022).

### 2.2 Behavioral analysis

Agonistic interactions were analyzed using QvodPlayer software (Version 5.0.80, Shenzhen Qvod Technology Co., Ltd.). We only analyzed individuals involved in boxing displays (see definition in Section 1). To avoid experimental bias caused by the effect of body orientation on boxing displays, only contests characterized by frontal body orientation were considered for further analysis. Hand preference in the boxing action was assessed by observing which forearm was first raised to hit the rival on the head and/or thorax.

Given the possibility that each bat could switch between the use of the left or right hand during an interaction, for each bat we calculated a laterality index (LI = (R − L)/(R + L), where R and L are the number of times the right‐ and left‐hand was used, respectively; LI ranges from −1 to 1). A positive value of LI indicates right‐hand bias, a negative value indicates left‐hand bias, and a zero value indicates no bias. We identified a boxing movement when an individual swung with its wrist toward a combatant.

### 2.3 Morphological data

Body mass and forearm length of each experimental bat were obtained from Sun et al. (2019) and Zhang, Sun, Lucas, Gu, et al. (2022). Individuals in each trial were size‐matched with body mass values within 10% of each other and with the difference in the ratio of the forearm length divided by the average body length between the paired males <2% ± 1.2% (Zhang, Sun, Lucas, Feng, & Jiang, 2022; Zhang, Sun, Lucas, Gu, et al., 2022).

### 2.4 Statistical analyses

As noted above, data on bats from the Simao site were derived from two different studies (Sun et al., 2019; Zhang, Sun, Lucas, Gu, et al., 2022). We ran Pearson's chi‐square tests to examine whether the proportion of hand preference and the proportion of laterality index significantly differed between Simao bats from the two studies. We found no significant differences in either parameter between bats from the two studies (Pearson's chi‐square test: $\chi_{1}^{2}$ = 0.070, *p* = .791; $\chi_{1}^{2}$ = 0.682, *p* = .409, respectively). Therefore, we pooled these data sets for subsequent analysis.

We conducted the following statistical analysis for bats from Simao, Hekou and Hanzhong, respectively. Exact binomial probability tests were performed to compare the number of bats with a left‐forearm and a right‐forearm preference, and to compare the number of bats with a negative LI value and a positive LI value. We also used kernel density estimates of the frequency distribution of laterality indices to offer a potentially more robust estimate of the entire frequency distribution. The kernel density estimation is a nonparametric method that adopts a slipped peak function to fit the sample data and utilizes a continuous density curve to describe the distribution pattern of the variables. It does not involve setting a functional form and can include observed variability in the data set with a continuous curve.

We also used an exact binomial probability test to evaluate whether the winners tend to display a left‐forearm or a right‐forearm preference. Moreover, in order to further confirm whether forearm preference has an effect on fighting ability, an exact binomial probability test was used to test whether the losers tend to display a left‐forearm or a right‐forearm preference.

## 3 RESULTS

**FIGURE 1:**
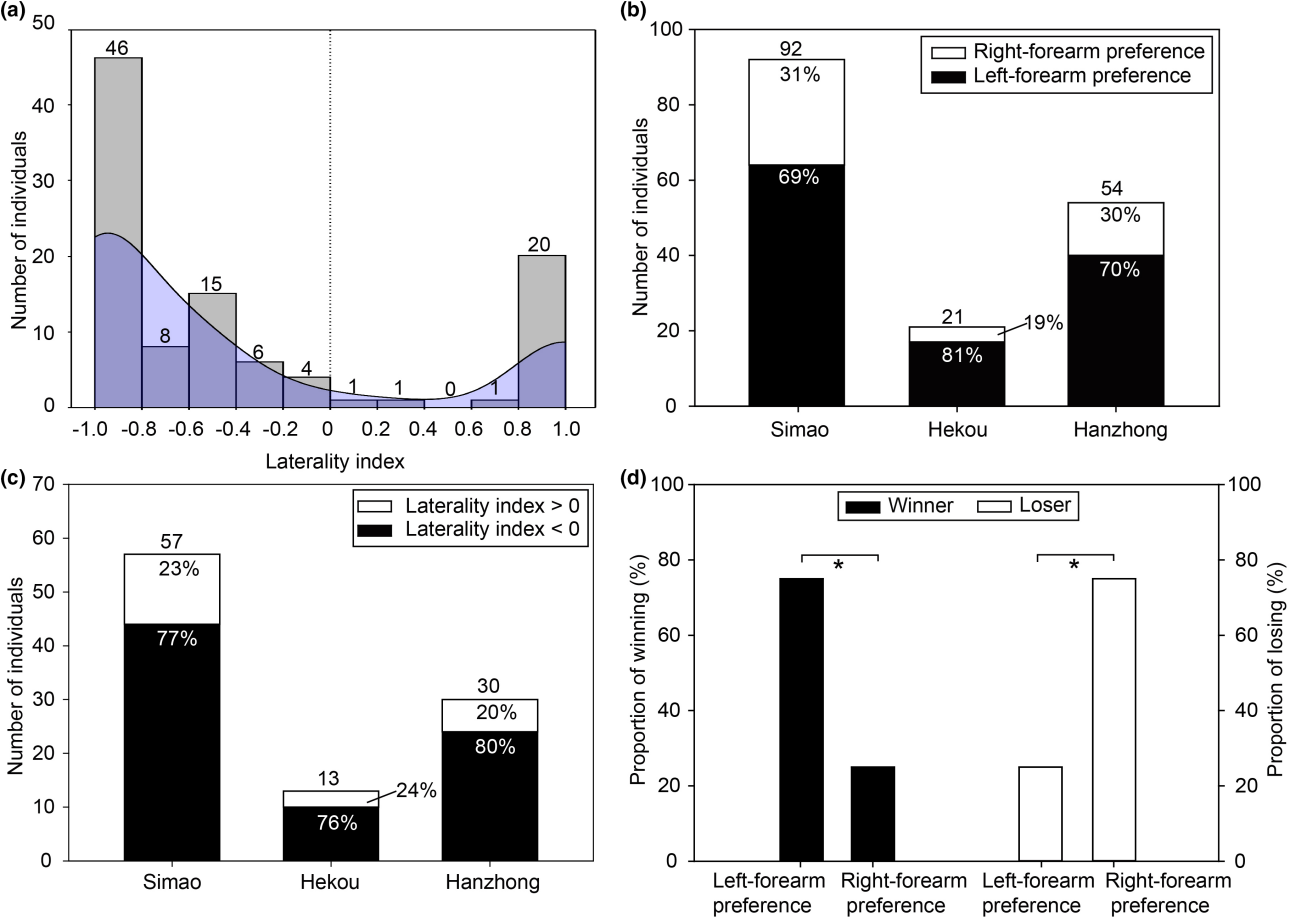
Forearm preference and fighting success in *Hipposideros armiger* contests. (a) Frequency histogram of laterality index (*N* = 102). Values to the left of zero indicate a left laterality, and values to the right indicate a right laterality. Vertical dotted lines indicate no laterality. Blue‐shaded area is kernel density estimates (see Section 2). (b) Number of individuals exhibiting left‐ and right‐forearm preference for Simao, Hekou, and Hanzhong populations. (c) Number of individuals with laterality index values greater or less than zero for three populations. The values above each histogram indicate the number of individuals. (d) The proportion of winning in winners and the proportion of losing in losers exhibiting left‐ and right‐forearm preference during an agonistic interaction. *p < .05.

A total of 167 individuals used boxing displays and we identified the winner or loser from 119 agonistic interactions (Simao: 59 contests; Hekou: 18 contests; Hanzhong: 42 contests; see Supplementary Material). The rest of the bats never struck their opponent. The number of boxing movements for the 167 males ranged from 1 to 15 (mean ± SD: 3.01 ± 2.73; Figure S1) with 102 males using at least two boxing movements. Of the 102 males, 36 (35%) switched the use of left and right hands and 66 (65%) did not switch. Of the 102 males, the mean ± standard deviation laterality index was −0.40 ± 0.77 (Figure 1a).

A significantly greater number of male *H. armiger* showed a left‐forearm preference in all three populations (binomial tests: Simao: *p* = .0002; Hekou: *p* = .0072; Hanzhong: *p* = .0005; Figure 1b). Except for the Hekou population (binomial tests: *p* = .092), the number of individuals with a negative LI value was higher than the number of individuals with a positive LI value (binomial tests: Simao: *p* = .0001; Hanzhong: *p* = .0033; Figure 1c).

Among the 119 fights, we only analyzed 20 interactions that involved size‐matched opponents and involved boxing displays by both opponents. A majority of winners were more likely to exhibit a left‐forearm preference than a right‐forearm preference (binomial test: *p* = .041; Figure 1d). On the contrary, most losers had more right‐forearm preferences than left‐forearm preferences (binomial test: *p* = .041; Figure 1d).

## 4 DISCUSSION

In this study, we found that the proportion of left‐handed boxers was significantly higher than the proportion of right‐handed boxers, which supported our first hypothesis that male *H. armiger* show a population‐level lateralized aggressive display. We also found that minority‐type males (right‐handed boxers) were less likely to succeed in fights than majority‐type males (left‐handed boxers), which failed to support our second hypothesis that minority‐type males will play a dominant role in conflict resolution (Ghirlanda et al., 2009).

We found that there is a left‐biased population‐level lateralization of aggressive behaviors: Male *H. armiger* displayed boxing preferentially with the left forearm. This result is consistent with the evidence that behavioral lateralization at the population level is more likely to evolve in social species since it may provide an underlying social function to group‐level behaviors (Deckel, 1995; Rogers et al., 2016). *H. armiger* is a highly social species, and frequent agonistic interactions among conspecifics during contests occurring in roosting space can be observed in day roosts (Sun et al., 2019; Yang, 2011). Population‐level lateralized boxing displays in male *H. armiger* may serve as a component of resource‐holding potential (Arnott & Elwood, 2009). Indeed, males with left‐lateralized aggressive displays were more likely to win a contest.

Why did male *H. armiger* show a preferential use of the left forearm? One possible interpretation is that social animals may have a strong lateral preference to use left body parts due to the preeminence of the right hemisphere in aggressive interactions (Bisazza et al., 1998). There is also behavioral evidence that the presence of a right‐side bias in the brain relative to the control of aggression is often linked to a preferential use of left body parts in aggressive interactions in a range of social animals including Mediterranean fruit flies, *Ceratitis capitata* (Benelli, Donati, et al., 2015), Australian stingless bees, *Tetragonula carbonaria* (Rogers & Frasnelli, 2016), red mason bees, *Osmia bicornis* (Rogers et al., 2016), Magellanic penguins, *Spheniscus magellanicus* (Stor et al., 2019), and Przewalski horses, *Equus przewalskii* (Austin & Rogers, 2014).

Contrary to the prediction of the competition–coordination model, we found that minority‐type males with a right‐forearm preference were less likely to achieve fighting success. Similar results can be found in blowflies *Calliphora vomitoria* (Romano et al., 2015), Mediterranean fruit flies *Ceratitis capitata* (Benelli, Donati, et al., 2015), and olive fruit flies *Bactrocera oleae* (Benelli, Romano, et al., 2015). This result is not particularly surprising because male *H. armiger* have been shown to make decisions in physical contests by assessments of their own ability (self‐assessment) rather than of their combatant's relative ability (mutual assessment; Sun et al., 2019). Therefore, it is reasonable to suggest that the unexpected fighting behaviors with a right‐forearm preference by an opponent would not affect an individual's decision to persist or to give up in a contest.

In summary, our study demonstrated that male *H. armiger* shows a population‐level lateralized aggressive display with a left‐forearm bias when using forearms for fighting. Moreover, left‐handed boxers won more contests than right‐handed boxers. To our knowledge, this is the first experimental evidence of lateralization of aggressive displays in a bat species. Further studies are needed to determine whether majority‐type males (i.e., left‐handed boxers) have a fitness advantage during intraspecific coordinated displays such as mating interactions.

## AUTHOR CONTRIBUTIONS

**Chunmian Zhang:** Conceptualization (equal); data curation (equal); formal analysis (equal); investigation (equal); methodology (lead); writing – original draft (lead). **Jeffrey R. Lucas:** Writing – review and editing (lead). **Jiang Feng:** Writing – review and editing (lead). **Tinglei Jiang:** Funding acquisition (equal); writing – review and editing (equal). **Congnan Sun:** Conceptualization (equal); data curation (equal); funding acquisition (equal); project administration (lead); writing – review and editing (equal).

## FUNDING INFORMATION

This research was supported by the Doctoral Research Foundation of Hebei Normal University (Grant nos 13116116) and the National Natural Science Foundation of China (Grant nos. 31922050, 31872680).

## CONFLICT OF INTEREST STATEMENT

The authors declare no competing interests.

## Supporting information


Figure S1

Video S1

Video S2

Table S1

Appendix S1

## Data Availability

Data are available in the Dryad Digital Repository: https://doi.org/10.5061/dryad.8gtht76t0.
