# Supplementary figures and images for: Population‐level lateralization of boxing displays enhances fighting success in male Great Himalayan leaf‐nosed bats
Source: Ecol Evol. 2023 Mar 8;13(3):e9879. doi: 10.1002/ece3.9879 (PMC9994608; doi:10.1002/ece3.9879)

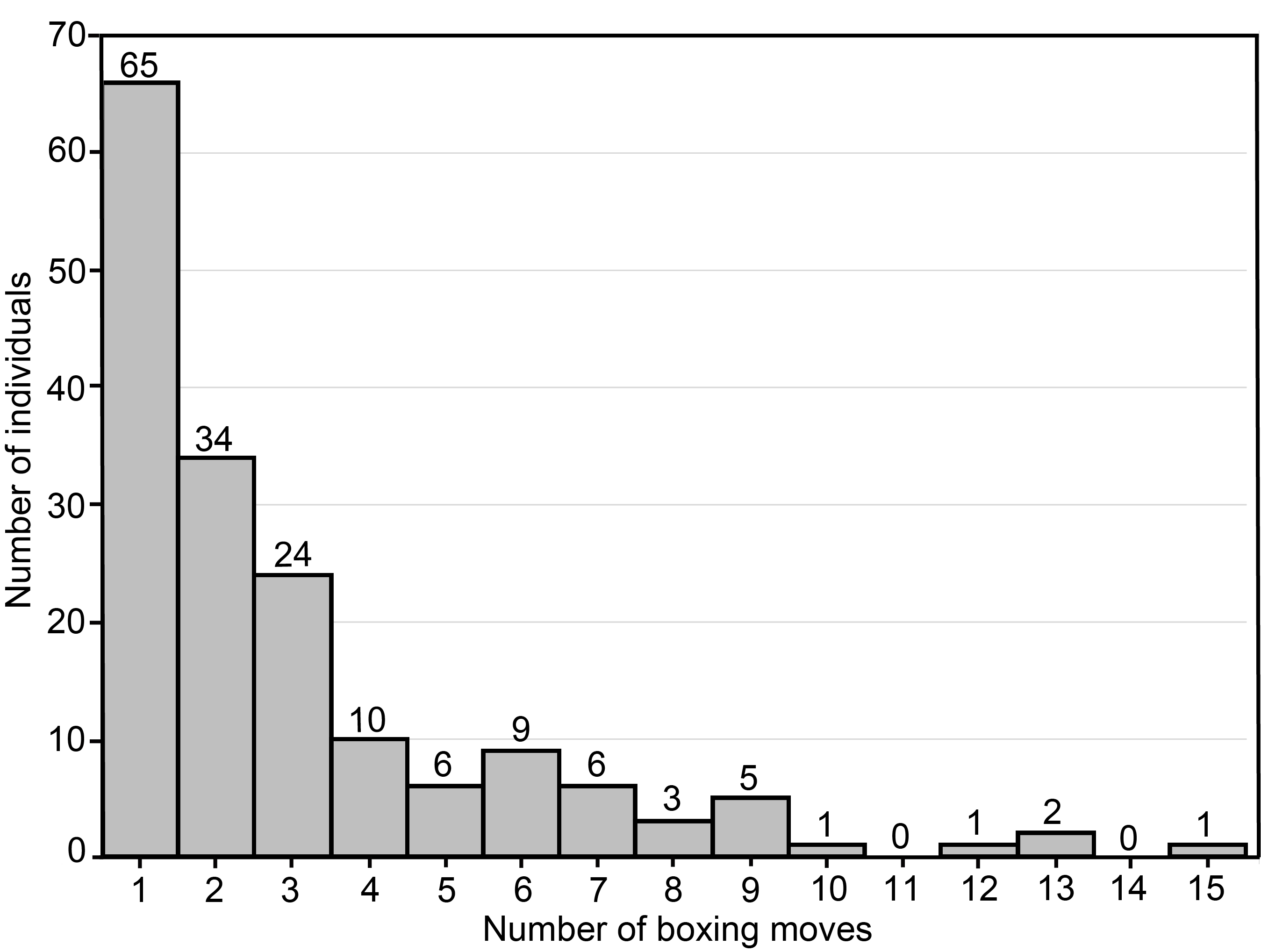

Supplement: Supplementary file 1 — Figure S1 [file ECE3-13-e9879-s003.jpg]
