# Supplementary material for: Population‐level lateralization of boxing displays enhances fighting success in male Great Himalayan leaf‐nosed bats
Source: Ecol Evol. 2023 Mar 8;13(3):e9879. doi: 10.1002/ece3.9879 (PMC9994608; doi:10.1002/ece3.9879)
Supplement: Supplementary file 5 — Appendix S1 [file ECE3-13-e9879-s005.docx]

**Electronic supplementary material**

**Figure S1** The frequency histogram of boxing moves during agonistic interaction for *Hipposideros armiger* (*N* = 167). The values above each histogram indicate the number of individuals.

**Video S1** Video of male *Hipposideros armiger* during agonistic interactions in Zhang et al. (2022a).

**Video S2** Video of male *Hipposideros armiger* during agonistic interactions in Sun et al. (2019).

**Table S1** Body size, number of boxing, laterality index of bats from three populations in China.
